# Supplementary figures and images for: ECS1 and ECS2 suppress polyspermy and the formation of haploid plants by promoting double fertilization
Source: eLife. 2023 Jul 25;12:e85832. doi: 10.7554/eLife.85832 (PMC10421590; doi:10.7554/eLife.85832)

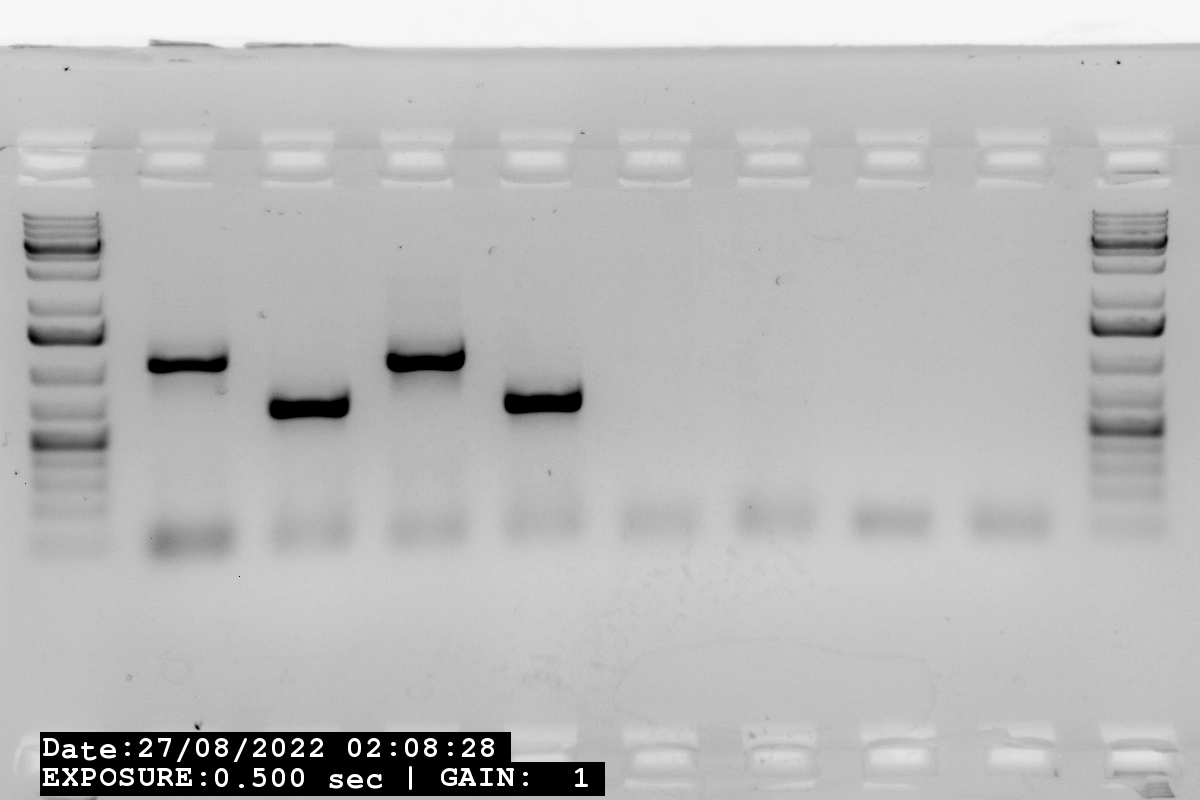

Supplement: Figure 1—source data 1. [file elife-85832-fig1-data1.zip › Figure 1-source data 1/Figure 1E-souce data unlabelled.tiff]

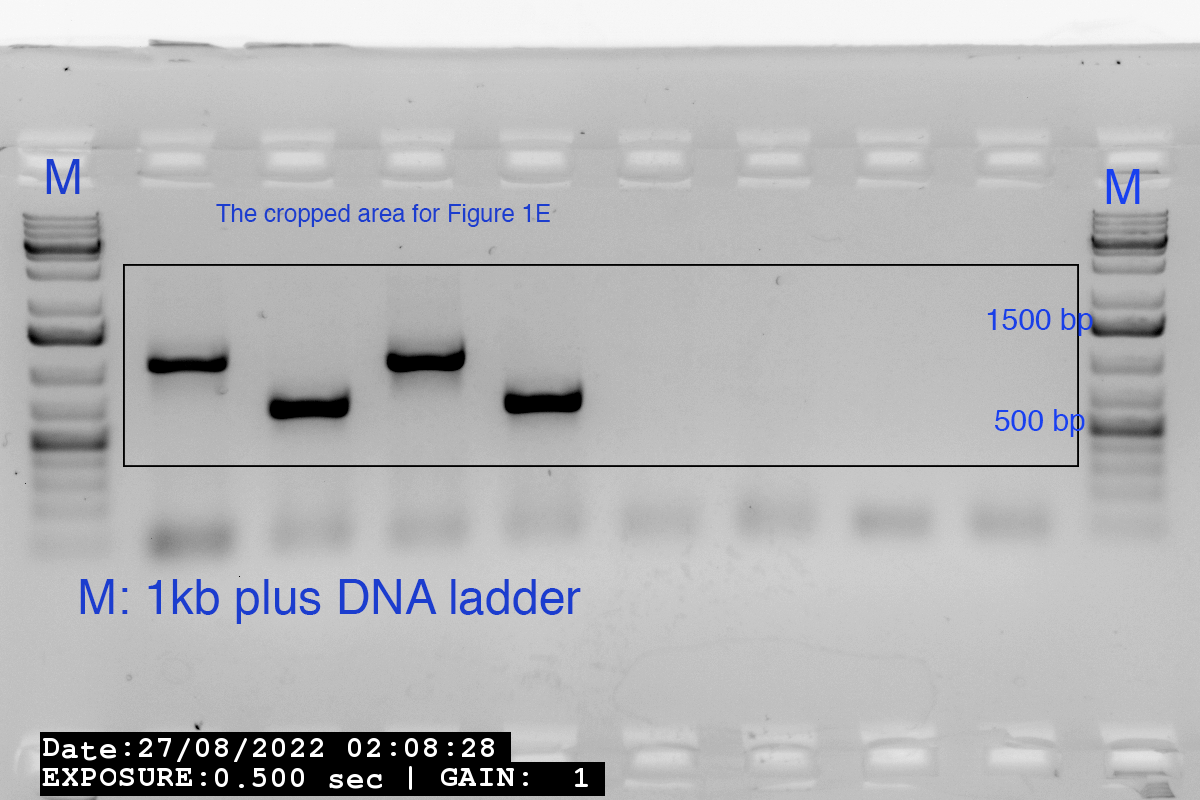

Supplement: Figure 1—source data 1. [file elife-85832-fig1-data1.zip › Figure 1-source data 1/Figure 1E-souce data labelled.tif]

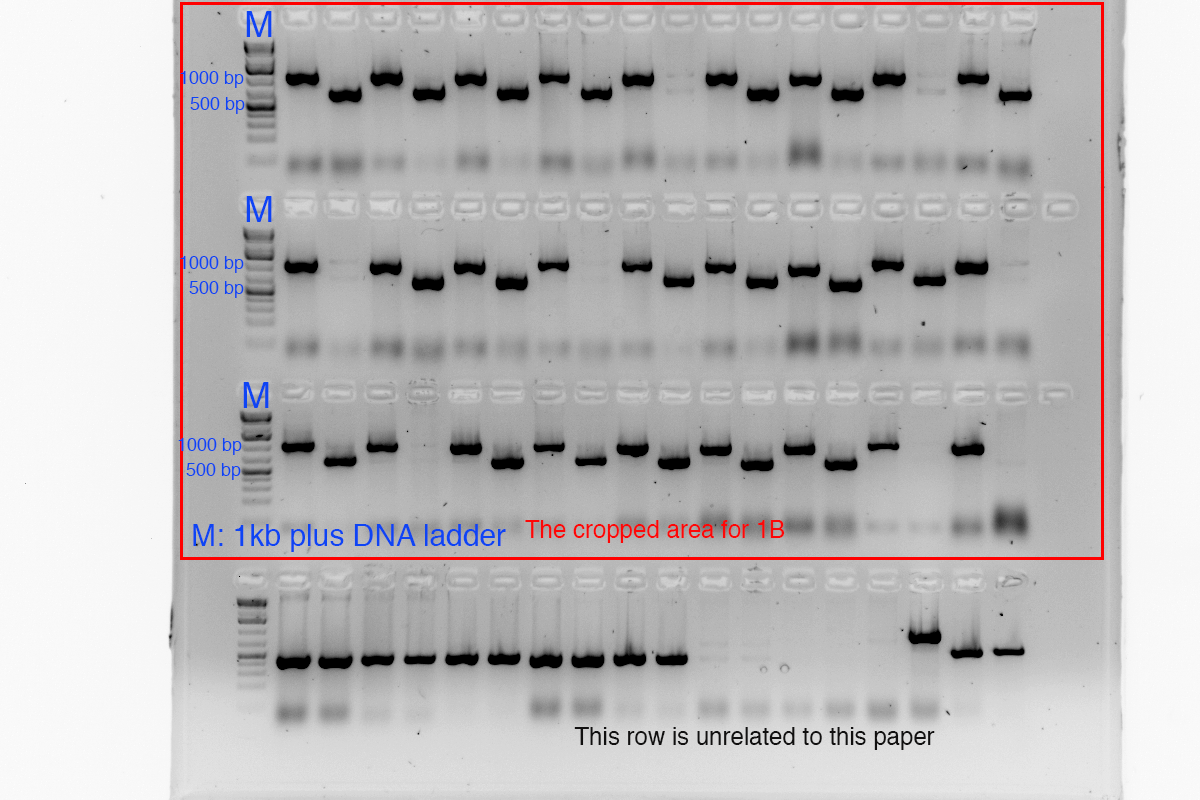

Supplement: Figure 1—figure supplement 1—source data 1. [file elife-85832-fig1-figsupp1-data1.zip › Figure 1- figure supplement 1-source data 1/1B-Source data labelled.tif]

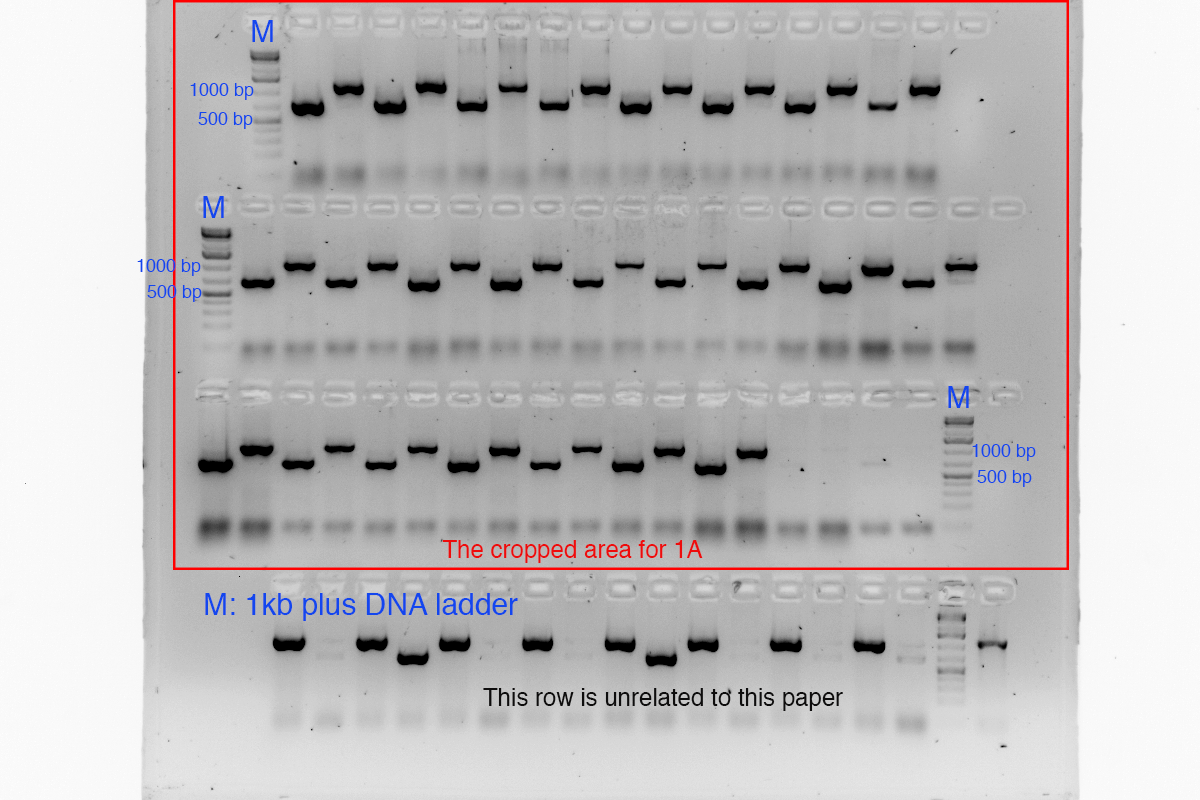

Supplement: Figure 1—figure supplement 1—source data 1. [file elife-85832-fig1-figsupp1-data1.zip › Figure 1- figure supplement 1-source data 1/1A-Souce data labelled.tif]

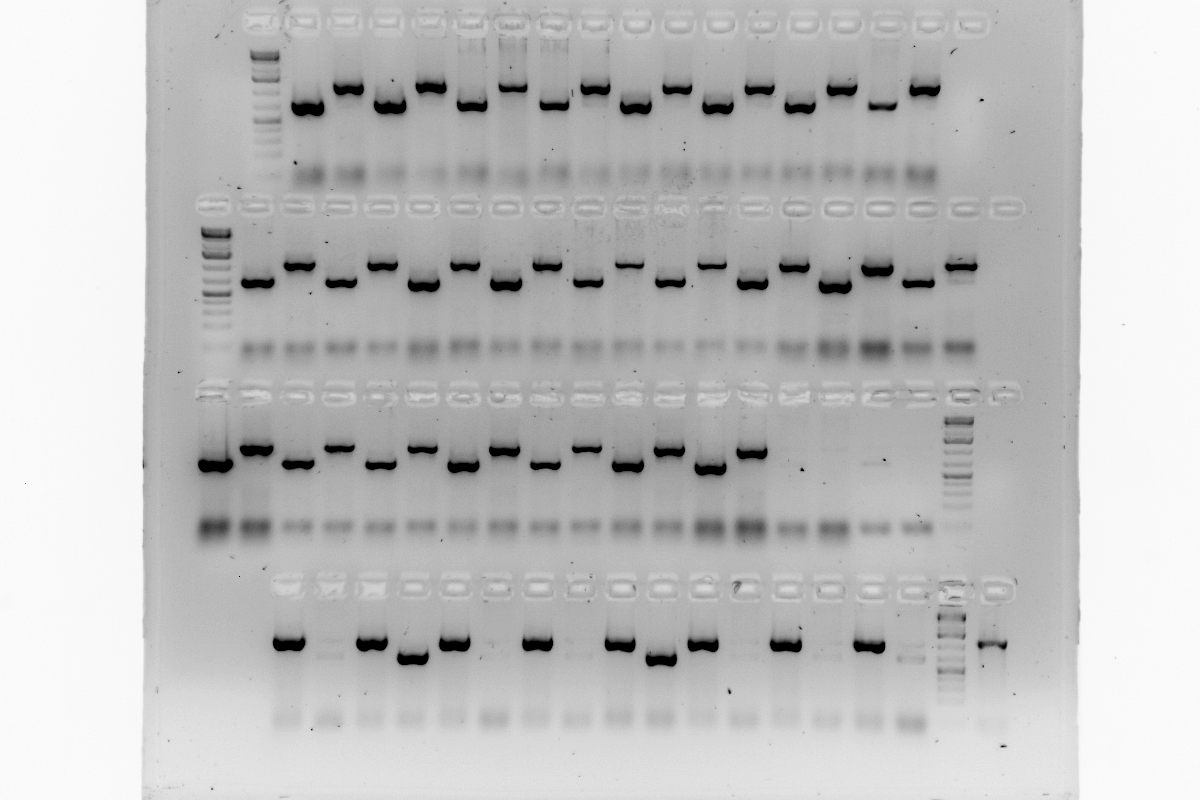

Supplement: Figure 1—figure supplement 1—source data 1. [file elife-85832-fig1-figsupp1-data1.zip › Figure 1- figure supplement 1-source data 1/1A-Souce data unlabelled.tiff]

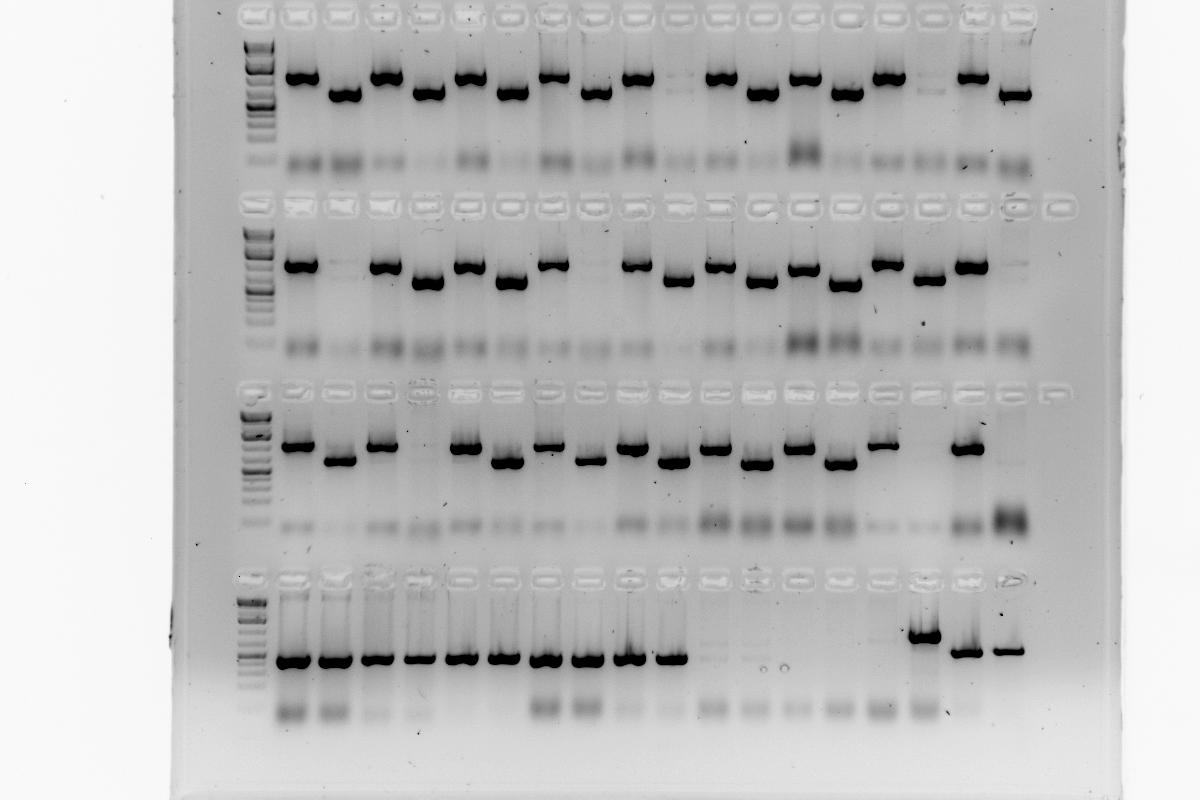

Supplement: Figure 1—figure supplement 1—source data 1. [file elife-85832-fig1-figsupp1-data1.zip › Figure 1- figure supplement 1-source data 1/1B-Source data unlabelled.tiff]

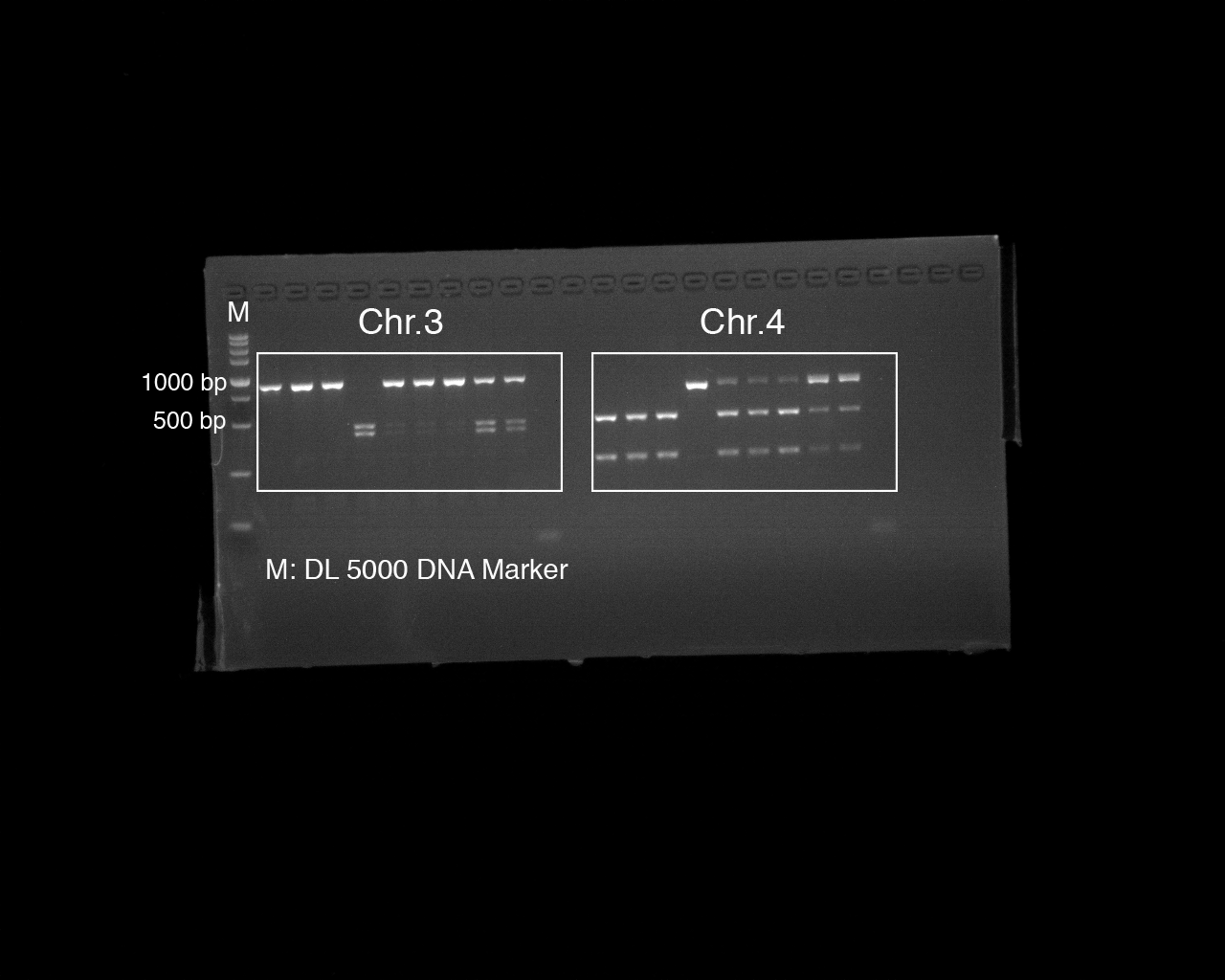

Supplement: Figure 4—figure supplement 3—source data 1. [file elife-85832-fig4-figsupp3-data1.zip › Figure 4-figure supplement 3- source data 1/Chr 3 and Chr 4 labelled.tif]

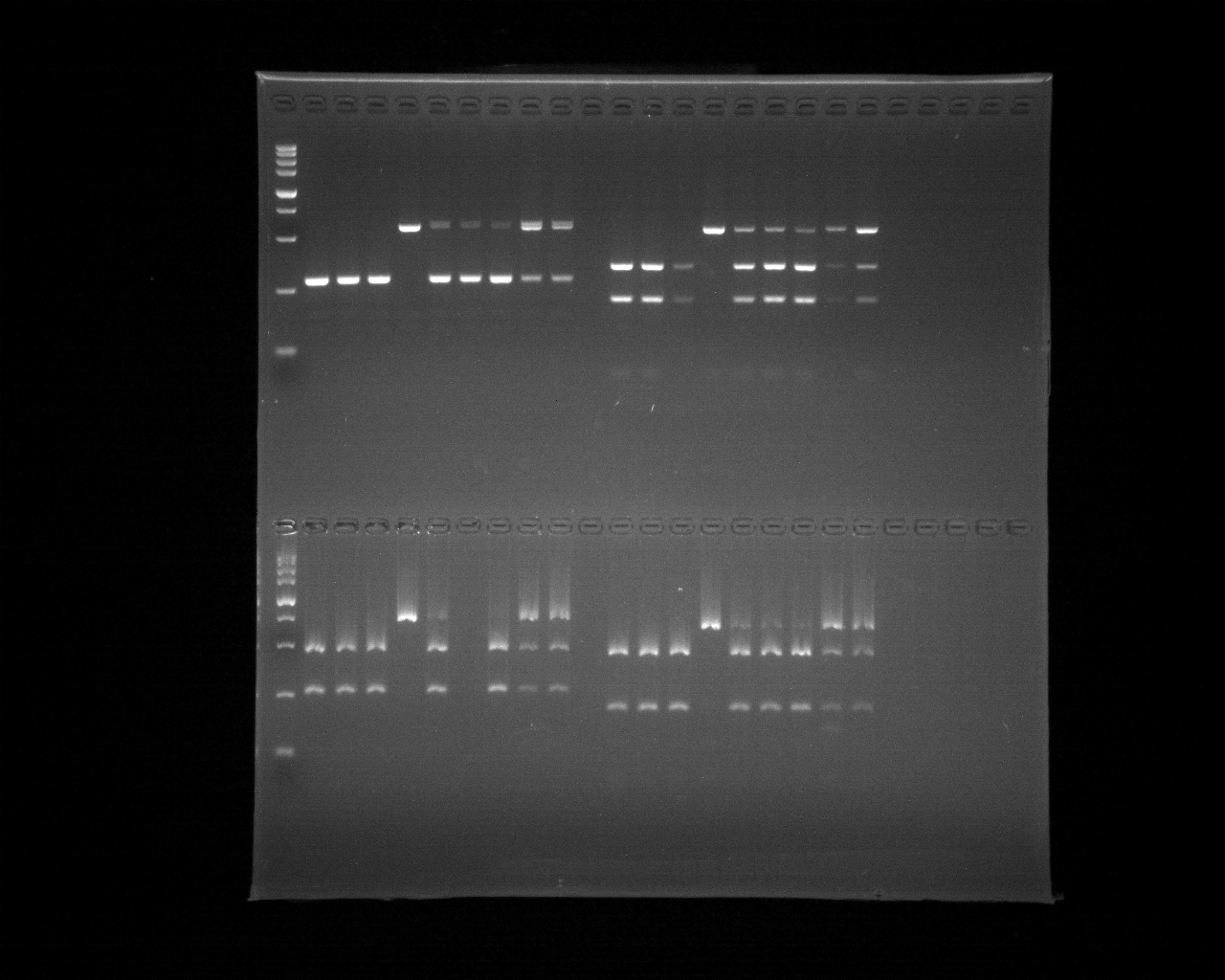

Supplement: Figure 4—figure supplement 3—source data 1. [file elife-85832-fig4-figsupp3-data1.zip › Figure 4-figure supplement 3- source data 1/Chr 1 and Chr 2 unlabelled.Tif]

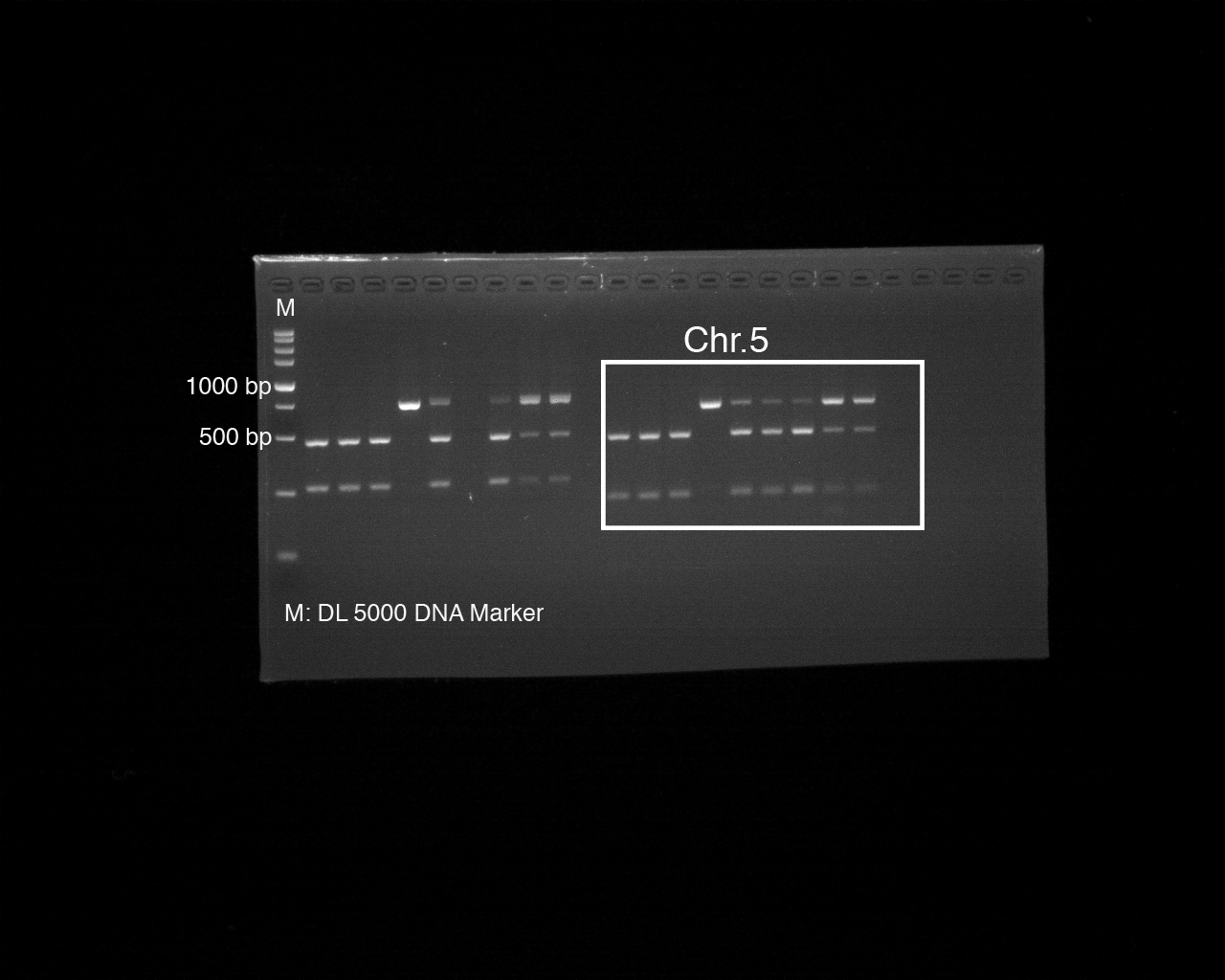

Supplement: Figure 4—figure supplement 3—source data 1. [file elife-85832-fig4-figsupp3-data1.zip › Figure 4-figure supplement 3- source data 1/Chr 5 labelled.tif]

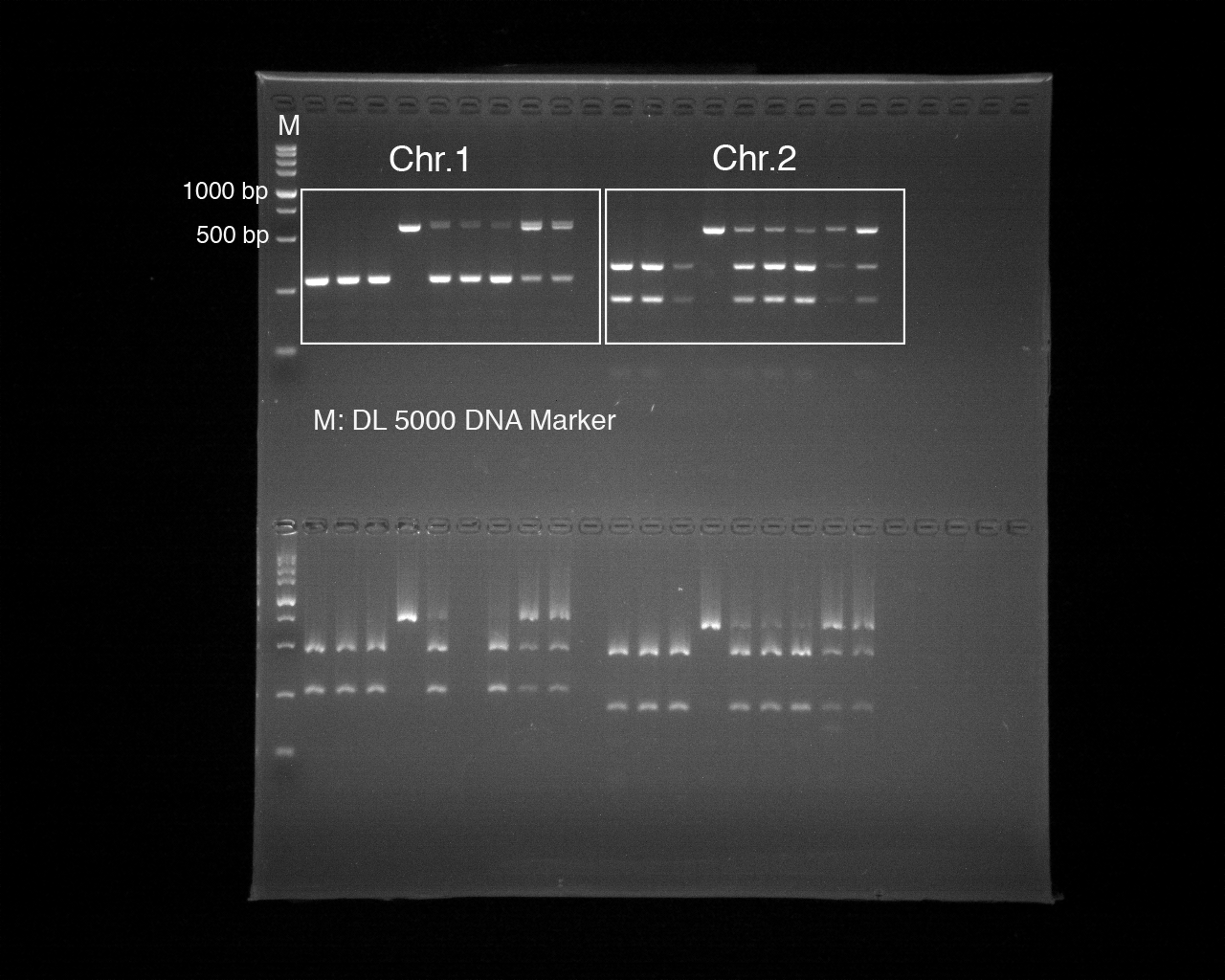

Supplement: Figure 4—figure supplement 3—source data 1. [file elife-85832-fig4-figsupp3-data1.zip › Figure 4-figure supplement 3- source data 1/Chr 1 and Chr 2 labelled.tif]

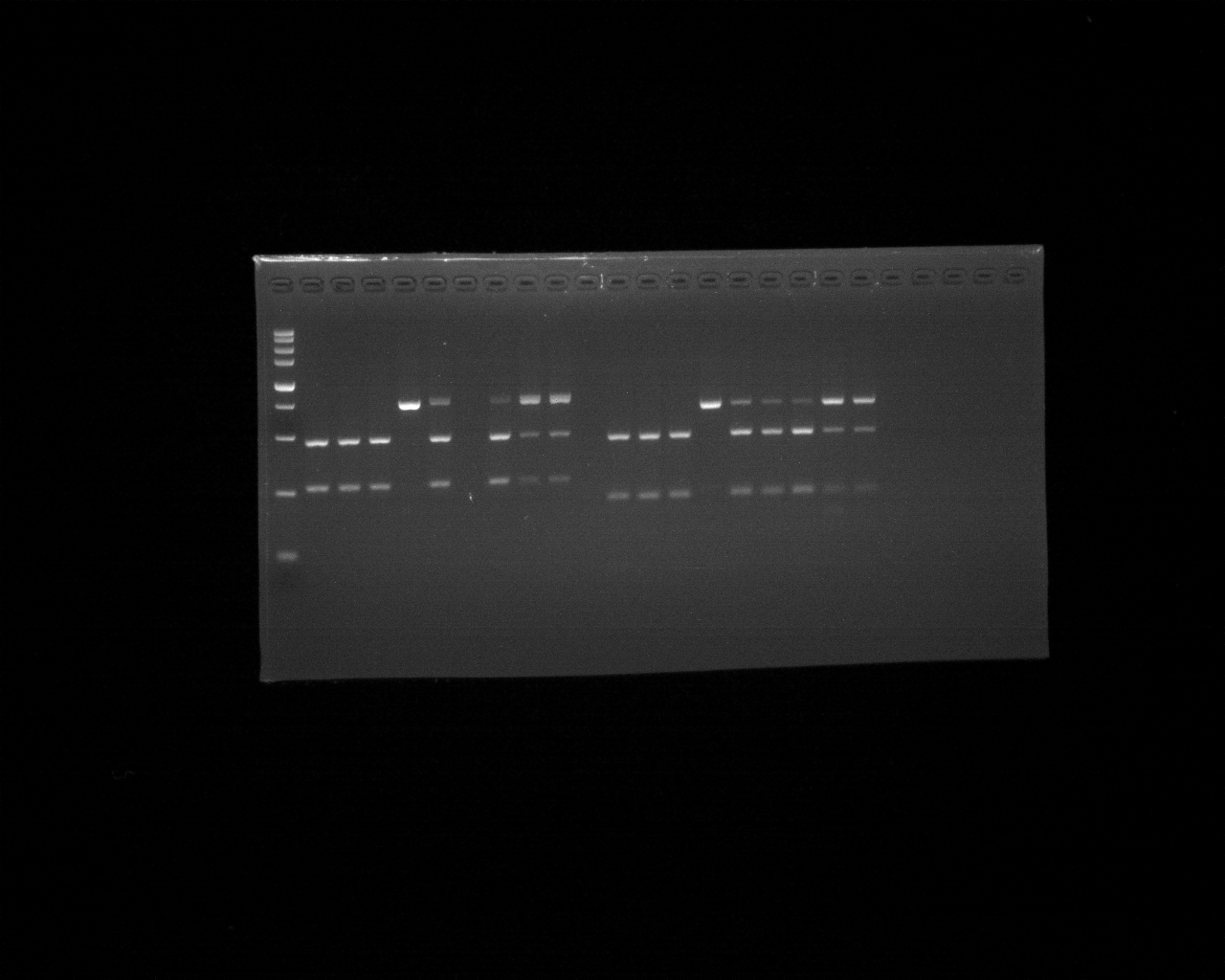

Supplement: Figure 4—figure supplement 3—source data 1. [file elife-85832-fig4-figsupp3-data1.zip › Figure 4-figure supplement 3- source data 1/Chr 5 unlabelled.Tif]

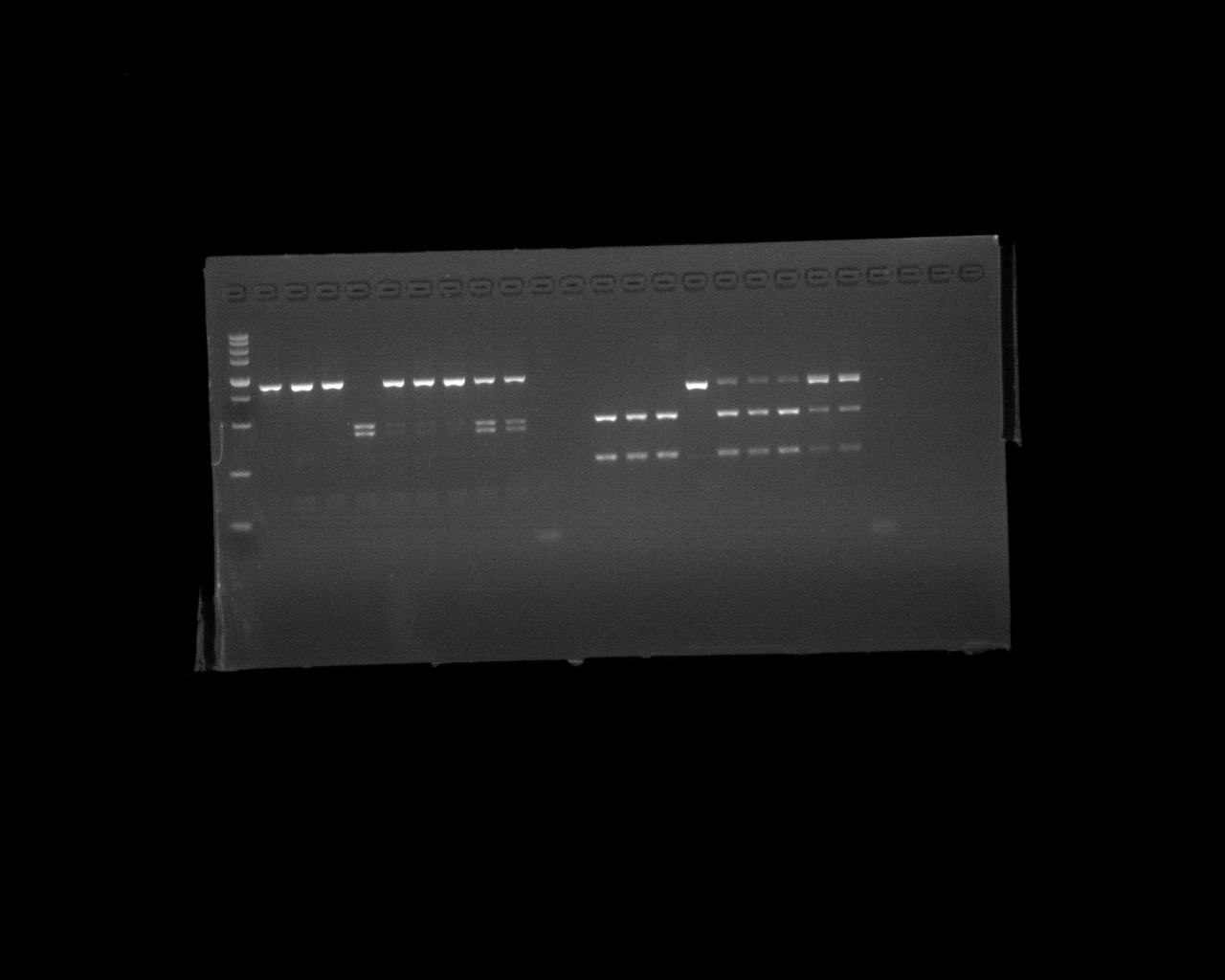

Supplement: Figure 4—figure supplement 3—source data 1. [file elife-85832-fig4-figsupp3-data1.zip › Figure 4-figure supplement 3- source data 1/Chr 3 and Chr 4 unlabelled.Tif]
